# Multi-Touch Tabletop System Using Infrared Image Recognition for User Position Identification

**DOI:** 10.3390/s18051559

**Published:** 2018-05-14

**Authors:** Shota Suto, Toshiya Watanabe, Susumu Shibusawa, Masaru Kamada

**Affiliations:** 1 Graduate School of Science and Engineering, Ibaraki University, Hitachi, Ibaraki 316-8511, Japan; macnamas@yahoo.co.jp; 2 East Japan Institute of Technology Co., Ltd., Hitachi, Ibaraki 319-1221, Japan; 3 National Institute of Technology, Gunma College, Maebashi, Gunma 371-8530, Japan; t.wat@ice.gunma-ct.ac.jp; 4 Hitachi Campus, Ibaraki University, Hitachi, Ibaraki 316-8511, Japan; 5 College of Engineering, Ibaraki University, Hitachi, Ibaraki 316-8511, Japan; masaru.kamada.snoopy@vc.ibaraki.ac.jp

**Keywords:** tabletop system, user position identification, infrared image recognition, multi-touch gesture, FTIR panel, system usability

## Abstract

A tabletop system can facilitate multi-user collaboration in a variety of settings, including small meetings, group work, and education and training exercises. The ability to identify the users touching the table and their positions can promote collaborative work among participants, so methods have been studied that involve attaching sensors to the table, chairs, or to the users themselves. An effective method of recognizing user actions without placing a burden on the user would be some type of visual process, so the development of a method that processes multi-touch gestures by visual means is desired. This paper describes the development of a multi-touch tabletop system using infrared image recognition for user position identification and presents the results of touch-gesture recognition experiments and a system-usability evaluation. Using an inexpensive FTIR touch panel and infrared light, this system picks up the touch areas and the shadow area of the user’s hand by an infrared camera to establish an association between the hand and table touch points and estimate the position of the user touching the table. The multi-touch gestures prepared for this system include an operation to change the direction of an object to face the user and a copy operation in which two users generate duplicates of an object. The system-usability evaluation revealed that prior learning was easy and that system operations could be easily performed.

## 1. Introduction

Hand gestures are a natural form of human communication and are seen as a promising means of human–computer interaction [1,2]. A tabletop system that allows for direct-touch input enables input by both hands in a natural and smooth manner surpassing conventional mouse and keyboard devices. It is expected to decrease the user’s cognitive load in interacting with content [3]. In small meetings and group work, a tabletop system is expected to provide an environment conducive to collaborative work. That is, with face-to-face interactions in which multiple users gather around a table, we can expect such a system to improve the contributions of each user and generate a sense of teamwork [4,5].

There have been many research studies to date on tabletop systems, including touch screen technology [6], a method for achieving an interactive table [7], research on multi-touch gestures [8,9], and applications for collaborative work support [4,5,10]. Among the methods used to achieve an interactive table, the frustrated total internal reflection (FTIR) method can obtain infrared images through a simple and inexpensive mechanism that irradiates the interior of an acrylic panel with infrared light and detects leaked light from the section of the panel touched by the user operating an infrared camera [7].

In the case of a multi-touch table used for simultaneous interactions by multiple users, being able to identify the users who are touching the table and their positions can facilitate group work, education and training [4], medical training and treatment planning [11], security control for machines, games, etc., that take participants into account. Suto et al. [12] presented a multi-touch tabletop system to identify user position using an infrared camera. By performing background differencing on the captured infrared images when a user performs a touch operation on the system, the tabletop image can be classified into three types of areas: the touch areas, the hand area, and the background itself. By establishing an association between the touch areas and the hand area, the system estimates the position of the user touching the table and the touch gesture.

Existing approaches to identifying users fall into three categories [13]: (1) approaches that augment the tabletop with additional sensors [14]; (2) approaches that require the user to wear or hold external sensors [15]; and (3) approaches that augment the objects surrounding the tabletop with sensors [16,17,18]. The technologies of the first approach are not yet mature. The second approach requires time to install those sensors and a step for learning how to use them, thereby sometimes placing the burden on the user. The third approach decreases the burden on the user to wear sensors, although it may place a few constraints on user position or posture.

A vision-based method following the third approach that detects parts of a user’s body and recognizes user actions outperforms the other methods in terms of ease of human motion and flexibility of system development. Suto’s tabletop system [12] has a feature to detect the user position and multi-touch gestures by a vision-based approach with one infrared camera.

In this paper, we describe a multi-touch tabletop system using infrared image recognition for user position identification that expands upon the previous system configuration and software we created [12,19]. We also present the results of touch-gesture recognition accuracy experiments and a system-usability evaluation. Using an inexpensive FTIR touch panel and a set of infrared lights placed above the FTIR panel, the infrared camera obtains information on table touch areas and the shadow area of the hand when a user performs a touch operation. By performing background differencing on the captured infrared images, the system establishes an association between the hand area and touch points and estimates the position of the user touching the table and the multi-touch gesture. The multi-touch gestures prepared for this system include an operation to change the direction of an object to face the user and a copy operation in which two users generate copies of an object in addition to basic touch gestures. The system-usability evaluation was conducted on the basis of a questionnaire based on the System Usability Scale (SUS) evaluation method [20], to which we have added a section for open comments.

This tabletop system has a feature to detect the user position and multi-touch gestures by a vision-based approach with one infrared camera. However, it is necessary to move the infrared light according to the tabletop move, and occlusion may arise according to user’s posture.

The rest of this paper is organized as follows. Section 2 summarizes related research on tabletop systems, Section 3 describes the proposed system and the user-position estimation technique, Section 4 describes an actual implementation of the system, Section 5 presents and discusses the results of touch-gesture recognition accuracy experiments and a usability evaluation using the implemented system, and Section 6 summarizes this study and touches upon future issues.

## 2. Related Research 

This section describes previous research related to user collaboration support by tabletop and its application, tabletop sensing methods, multi-touch gestures, and user position identification.

### 2.1. User Collaboration Support by Tabletop and Its Application

Interaction among multiple users operating a tabletop system can be broadly divided into face-to-face interaction around a single table and distributed interaction around tables installed in different spaces. A tabletop system based on face-to-face interaction can help each user make a deeper contribution to the topic of discussion while promoting team bonding. Systems of this type are expected to be especially effective in educational activities for young people and the planning of medical treatments through interaction between medical personnel.

Morris et al. [4] used the software of the DiamondTouch table, capable of multi-user identification, to implement 11 gesture applications and study the use of cooperative gestures in multi-user interaction. Isenberg et al. [5] performed an exploratory study on co-located collaborative visual analytics around a tabletop display and confirmed that teams that worked closely together and communicated throughout were more successful at a given task and required fewer assists. Evans et al. [10], meanwhile, examined the relationship between touch interactions and the collaborative process in field studies of adolescent students and showed that touch patterns reflect the quality of collaboration. In addition, Ohashi et al. [21] constructed a computerized KJ method support system that enabled finger pointing and measured working time and the number of comments made, and showed that this system with finger pointing could reduce working time.

In the fields of clinical medical treatment and surgery, there are high hopes for practical technologies based on mixed reality that overlay information from diverse types of sensors with real-time images. The advancement of interaction technologies in these fields has therefore become a major issue [22]. Lundström et al. [11] developed a table system for medical visualization for orthopedic surgery planning and discussed issues in system design. They found that essential design objectives in the configuration of such a system that includes interaction are highly similar to actual physical conditions, providing a very low learning threshold.

Genest et al. [23] developed a toolkit called KinectArms to capture and display arm embodiments with the aim of facilitating gesture-driven communication in remotely distributed tables. KinectArms provides a visual representation of arms by using a depth camera to determine gesture height and improves the expressive power and usability of distributed tabletop groupware. For pairs of users performing collaborative tasks using tablets and tabletops, Zagermann et al. [24] studied the effect of the size of a shared tabletop on users’ attention, awareness, and efficiency and found that larger tabletops do not necessarily improve collaboration or sensemaking results.

### 2.2. Tabletop Sensing Methods

A number of touchscreen technologies have been developed to enable a person to manipulate a display screen through touch. These include projected capacitive, analog resistive, infrared, camera-based optical, planer scatter detection, vision-based, and combinations of technologies. Walker [6] presented a broad overview of 13 types of touchscreen technologies, describing for each a brief history, basic operating principle, typical applications, main advantages and disadvantages, current issues and trends, and future outlook. He described the projected capacitive method in more detail than the other methods due to its current dominance.

Han [7] described a detailed implementation of an FTIR-based multi-touch, interactive table and outlined the future direction of multi-touch sensing technology. Zhang et al. [25] introduced sensing technology that enables touch input on the surface of objects having irregular and complex forms using electric field tomography and demonstrated the feasibility of this technology using example applications.

### 2.3. Multi-Touch Gestures

Typical gestures on a tabletop include move, zoom in/out, rotate, drag, tap, flick, and hold. The tabletop user employs these gestures created by the system designer. These gestures, though thought to be appropriate in initial research, do not necessarily reflect user actions. Designing natural and intuitive gestures for a new multi-touch interface therefore requires a survey on how users would approach a multi-touch interface and the types of gestures they would use.

North et al. [26] asked users to execute an object-sorting task on a physical table, multi-touch surface, and desktop computer with a mouse, measured and compared task execution times, and collected the set of user gestures on the multi-touch surface. Wobbrock et al. [8] collected 1080 gestures for the case of 20 nonexpert users operating a tabletop with one and two hands, paired those gestures with 27 commands, and examined the way in which users employed multi-touch gestures. Hinrichs et al. [9] used a large multi-touch tabletop exhibited at a municipal aquarium to conduct a field survey on the use of multi-touch gestures by visitors. They found that the use of multi-touch gestures was influenced by user preference, usage conditions, and social conditions and that previous gestures influenced subsequent gestures to form gesture sequences.

### 2.4. User Position Identification

The ability to identify the users touching a multi-touch tabletop and their positions opens the door to diverse methods of use in collaborative group work and other scenarios. Dietz et al. [16] described the design method, construction, and usage results of DiamondTouch, a technology for identifying the positions of particular users touching a multi-user touch table from electric fields generated by capacitive coupling between the users and their chairs. Marquardt et al. [15] developed the TouchID toolkit for multi-touch tabletop interaction with fiduciary-tagged gloves and described its suite of techniques. This toolkit can gather information on the person touching the table, the hand being used, which hand part, and hand posture and gesture.

Annett et al. [14] created a tabletop system equipped with 138 proximity sensors around a Microsoft Surface to detect a user’s position, distinguish between left and right arms, and establish a correspondence between touch points, users, and hands. Lissermann et al. [17] created an environment supporting group work, individual work, and in-between transitions using a multi-view tabletop consisting of a multi-touch frame, 3D display, two Kinect cameras for user and hand recognition, and 3D shutter glasses worn by users. They described its implementation techniques and presented application examples. 

Zhang et al. [18] determined the contours of users’ hands with an infrared lamp above an FTIR table, predicted user position by machine learning based on the finger orientation distributions of users touching the tabletop surface, and measured the accuracy achieved. Their study does not refer to multi-touch gestures. Evans et al. [13] proposed a method to distinguish tabletop users in group settings using Microsoft PixelSense on-board cameras and performed a statistical analysis of wild data. Their method does not identify or track users.

Finally, Suto et al. [12,19] created a multi-touch tabletop system that identifies user position by image recognition using an FTIR touch panel and external infrared light. They investigated the accuracy of recognizing multi-touch gestures with this system.

## 3. System Configuration

### 3.1. System Overview

**Figure 1:**
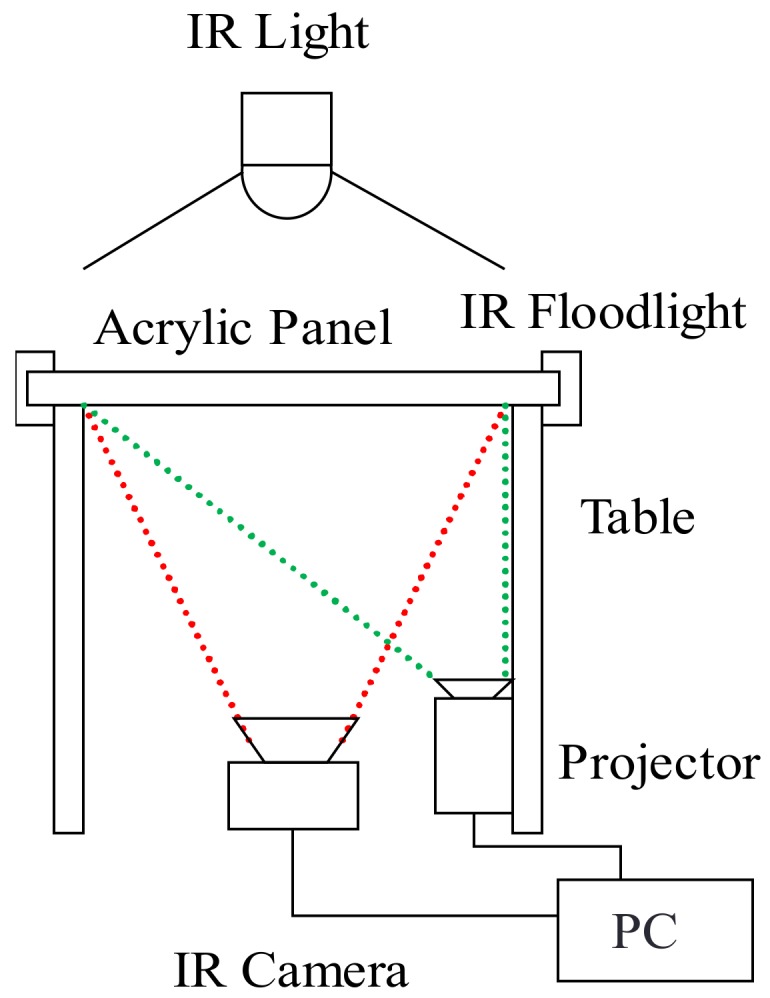
System configuration.

The basic configuration of a multi-touch tabletop system consisting of an FTIR table and infrared light is shown in Figure 1. This system installs an infrared camera underneath the table to capture the acrylic panel on top of the table and a projector connected to a personal computer (PC) to project images onto the panel. It installs an infrared floodlight consisting of an infrared LED on both ends of the acrylic panel for irradiating the panel with infrared light. In addition, the system pastes tracing paper that plays the role of a screen onto the acrylic panel that becomes the image-projection surface and presents information to users by projecting images from the projector.

Furthermore, to obtain information on a user’s hand area, the system installs an infrared light on the ceiling above the table. Since a user’s hand on the tabletop will obstruct infrared beams from this light, an infrared shadow corresponding to the area of the hand will form, enabling the shadow to be picked up by the infrared camera.

### 3.2. Overview of User-Position Estimation Technique

In a tabletop system, a user extends a hand from the edge of the table to manipulate an object displayed on the tabletop. At this time, the ability of determining from which direction the hand touching the object is being extended would make it possible to estimate the position of the user manipulating those touch points.

Touch points on the FTIR table appear as white light and the shadow of the user’s hand appears on the table owing to the overhead infrared light. The infrared camera picks up both of these images. Now, by performing background differencing to these captured images, the tabletop image can be classified into three types of areas: the touch areas having higher brightness values than the background, the hand area having lower brightness values than the background, and the background itself. Here, brightness value *b* of the captured tabletop image can be expressed as follows with respect to threshold values *σ*_1_, *σ*_2_ (*σ*_1_ < *σ*_2_):

(1) $$\begin{array}{cc} b<\sigma_{1} & \;\text{:}(\mathrm{Hand}\;\mathrm{Shadow})\\ \sigma_{1}\leq b<\sigma_{2} & \text{:}(\mathrm{Background})\\ b\geq \sigma_{2} & \text{:}(\mathrm{Touch}\;\mathrm{Area}) \end{array}\}$$

The union of the touch areas and the hand-shadow area constitutes an area having a change in brightness values with respect to the background. It can be defined as the hand area as follows:

(2) $$\left(b<\sigma_{1}\right)\cup^{\text{​}}\left(b\geq \sigma_{2}\right)\;\;\text{:}(\mathrm{Hand}\;\mathrm{Area})$$

**Figure 2:**
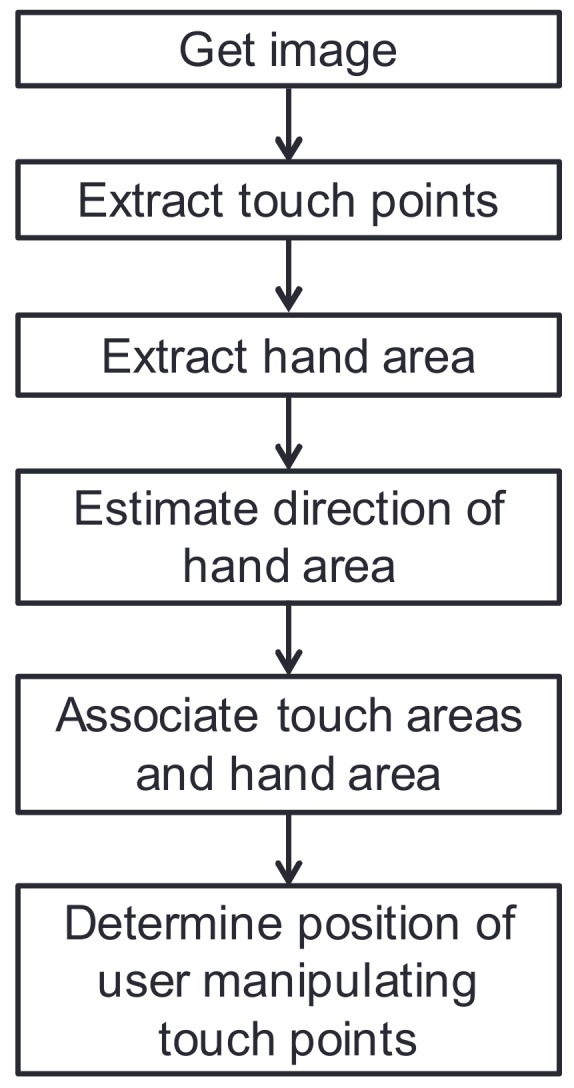
Procedure of estimating position of user manipulating touch points.

The procedure for estimating the position of the user manipulating the touch points is shown in Figure 2. In this process, the system separately extracts the touch points and hand area, superposes and associates these areas, and determines the position of the user manipulating the touch points. A detailed description of this process is described in the following two subsections.

**Figure 3:**
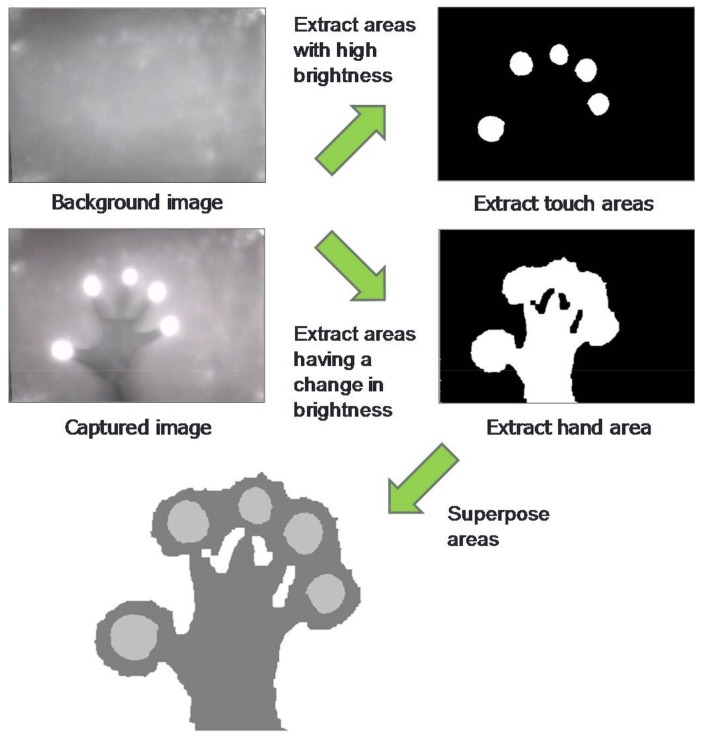
Extraction and superposition of key areas.

An example of extracting and superposing touch areas and the hand area is shown in Figure 3. The direction of the hand area is determined by the amount of shadow occupying edges of the tabletop. Given that the touch areas constitute a subset of the hand area, the position of the user manipulating the touch points can be estimated. In this example, the system would estimate the user associated with the touch points to be positioned downward relative to the figure.

### 3.3. User-Position Estimation Model

#### 3.3.1. Inclusive Relation between Touch Points and Hand Area

**Figure 4:**
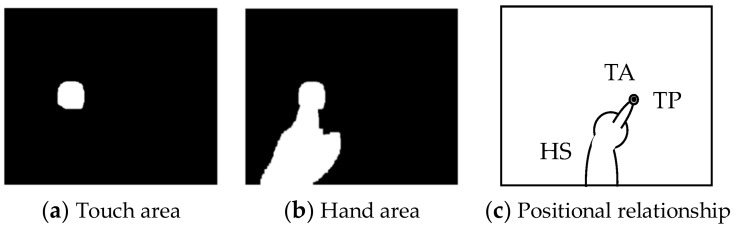
Images of touch area and hand area and their positional relationship.

In this FTIR system, the touch points are captured as white light and the hand shadow as an infrared shadow generated by the overhead infrared light. The touch areas and hand area can be extracted by performing background differencing on this image. An image of a touch area and that of a hand area including that touch area extracted by background differencing are shown in Figure 4a,b, respectively. In addition, the positional relationship among the touch area (*TA*), touch point (*TP*), and hand shadow (*HS*) is shown in Figure 4c. Here, the outer circle and inner circle at the fingertip corresponds to *TA* and *TP*, respectively. The *TP* is determined by calculating the center of gravity of *TA*.

Given that *TP* is the center of gravity of *TA*, the following relation describing *TP* as an element of *TA* holds:

(3) TP∈TA

Furthermore, as defined in Equation (2), hand-area *Hand* is the union of *TA* and *HS*.

(4) $$Hand\equiv TA\cup^{\text{​}}HS$$

From Equation (4), the following inclusive relations with respect to *Hand* and *TA*/*HS* hold:

(5) TA⊂Hand

(6) HS⊂Hand

Now, from Equations (3) and (5), the following relation holds describing *TP* as an element of *Hand*:

(7) TP∈Hand

Although the touch point and hand area are determined by different processing, Equation (7) shows how a certain touch point relates to a certain hand area.

#### 3.3.2. Model for Estimating User Position

The proposed technique first determines the correspondence between *TP* and *Hand*. It then determines from which edge in the image the area indicated by *Hand* is extending and defines that direction as the touch-point direction. The position of the user manipulating that touch point can be estimated in this way.

**Figure 5:**
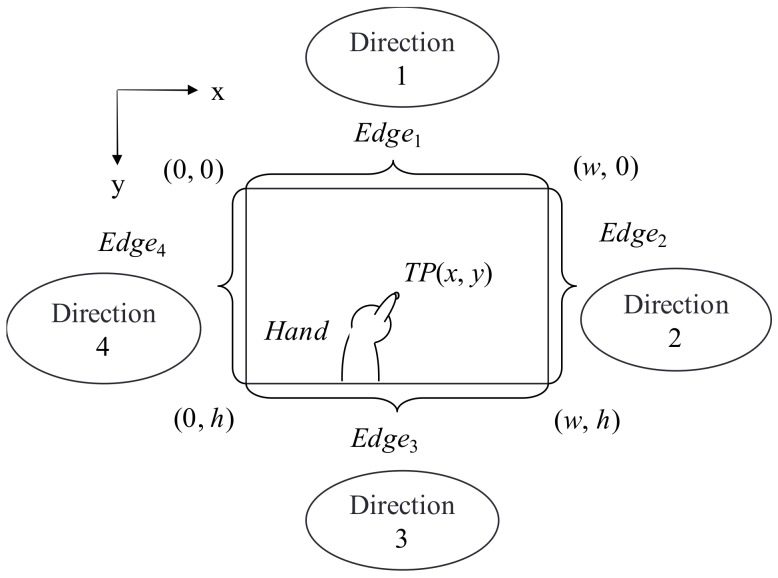
User position estimation model.

The model for estimating the position of the user associated with a certain touch point is shown in Figure 5. In the figure, the width and height of the image are denoted as *w* + 1 and *h* + 1, respectively, and the directions corresponding to the four edges of the tabletop are denoted as *d* (*d* = 1, 2, 3, 4). At this time, the coordinate set *Edge_d_* corresponding to direction *d* can be expressed as follows:

(8) $$\begin{array}{c} Edge_{1}=\{\left(i,0\right)|0\leq i\leq w\}\\ Edge_{2}=\{\left(w,j\right)|0\leq j\leq h\}\\ \begin{array}{c} Edge_{3}=\{\left(i,h\right)|0\leq i\leq w\}\\ Edge_{4}=\{\left(0,j\right)|0\leq j\leq h\} \end{array} \end{array}\}$$

Given the detection of hand-area *Hand*, the intersection of the edge coordinate sets and hand area exists, so the following condition holds:

(9) $$\;Hand\cap^{\text{​}}\left\{\overset{4}{\underset{d=1}{\cup }}Edge_{d}\right\}\neq \emptyset$$

### 3.4. User-Position Estimation Technique

Multiple hand areas usually exist on a multi-user tabletop. To recognize individual hand areas, this technique performs labeling with respect to areas having connected pixels and assigns the label *L*[*Hand*] to each hand-area *Hand*. It then assigns label *L*[*TP*(*x*, *y*)] to each touch-point *TP* in the areas determined by touch-point extraction. According to Equation (7), *Hand* includes *TP*(*x*, *y*), which means that *L*[*TP*(*x*, *y*)] has the same value as label *L*[*Hand*] of the hand area manipulating *TP*.

Referring to Figure 4b, *Hand* consists of a contiguous area that may be connected to more than one edge of the image. Accordingly, by investigating which edges *Hand* is actually connected to, the direction from which *Hand* is being extended can be determined. Specifically, by comparing the number of pixels of each edge to which the shadow area of that hand intersects with, the edge with the most intersecting pixels is taken to be the direction from which the hand is being extended. The following steps can be used to estimate the direction of user touch points:

1. Scan label *L*[*Edge_d_*] of each edge and calculate the number of pixels *Pixel_d_* having the same label as hand-label *L*[*Hand*].
2. Derive the value of *d* satisfying MAX[*Pixel_d_*], establish that *Hand* is extending from direction *d*, and infer that the direction of the position of the user manipulating *TP* is *d*.

**Figure 6:**
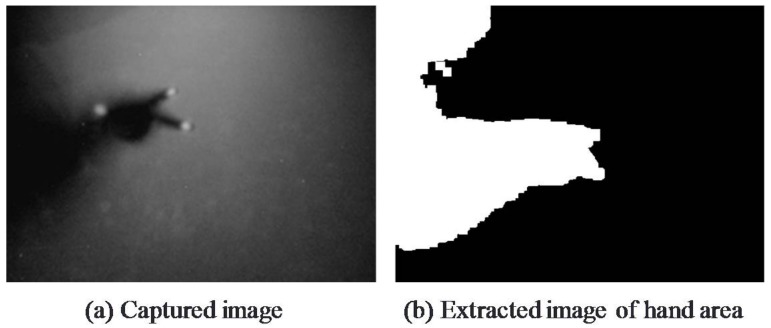
Extraction of hand-area image.

An example of an image of a hand area is shown in Figure 6. Specifically, Figure 6a,b show the captured image and the image of the extracted hand area, respectively. In this example, number of pixels *Pixel*_4_ of *Hand* on *Edge*_4_ takes on a maximum value, which means that *TP* will be taken to be the touch points manipulated by the user positioned in direction 4. We note here that the user in this example is peering down at the tabletop when making gesture operations. This posture results in the casting of a shadow of the user’s upper body on the table with the result that the hand area crosses multiple edges.

### 3.5. Object Touch Gestures

**Figure 7:**
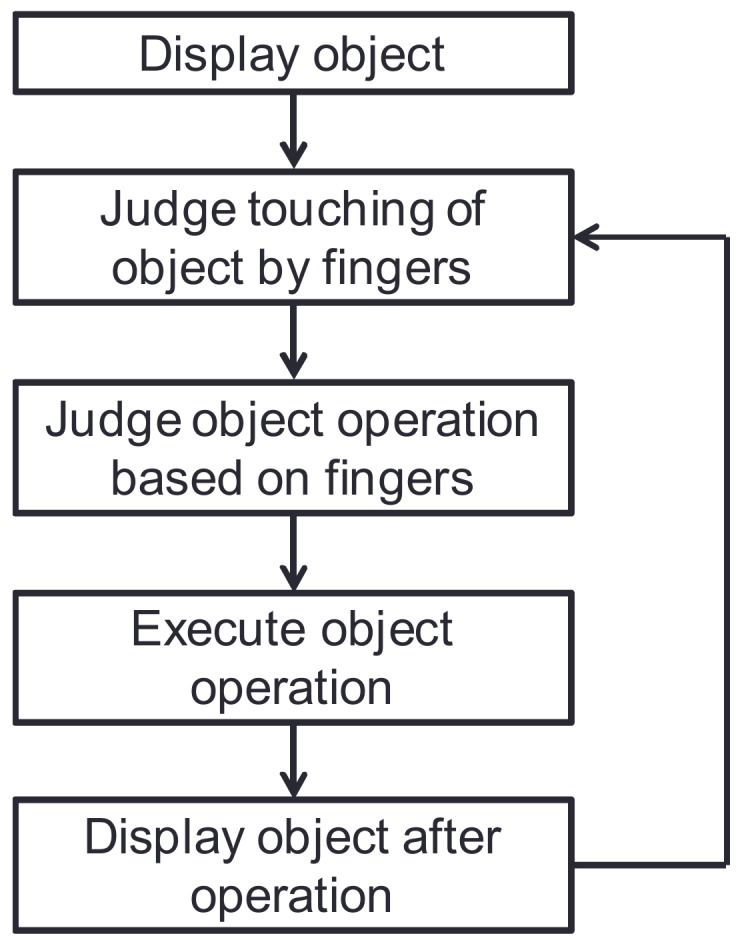
Procedure of object manipulation.

This system has a function for manipulating displayed objects through the use of multi-touch gestures. The procedure of object manipulation is shown in Figure 7. In this procedure, the system judges that fingers are touching an object displayed on the tabletop and determines the type of object operation based on the type of finger action. It then executes that object operation and redisplays the object. The touch gestures provided by this system are listed in Table 1 and described below.

**Figure 8:**
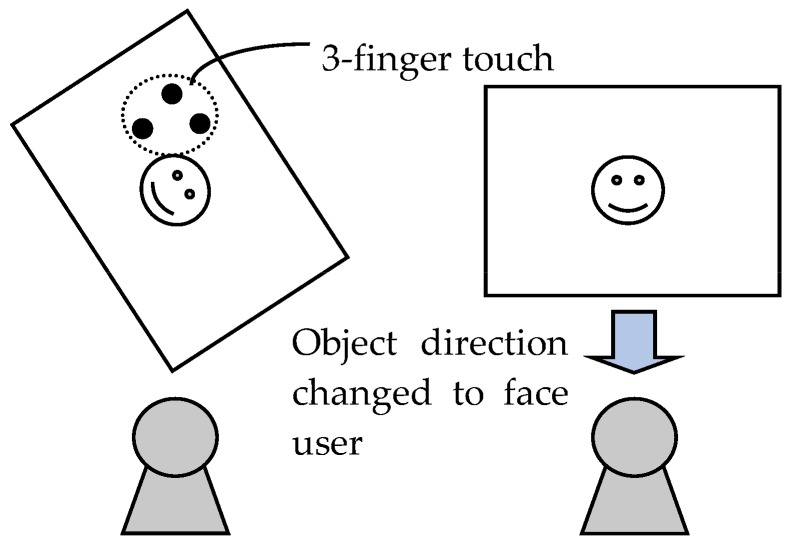
Change-direction gesture.

**Figure 9:**
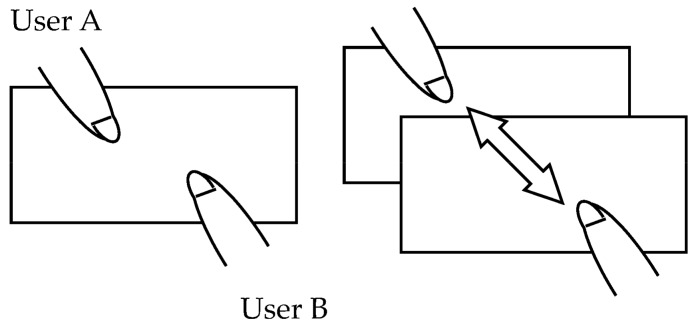
Copy gesture.

- Move object:
  With one finger touching the object, this gesture moves the object by moving the fingertip. The system detects finger movement and moves the object by only the amount of finger movement in the direction of that movement.
- Zoom object in/out:
  With two fingers touching the object, this gesture zooms the object in or out by expanding or contracting the space between the fingertips. The system detects the movement of these two fingers and expands the object if that space lengthens and contracts the object if that space shortens.
- Rotate object:
  With two fingers touching the object, this gesture rotates the object by performing a finger-twisting type of action. The system calculates the angle of rotation from the inclination of the two fingers and rotates the object accordingly.
- Change direction of object:
  With three fingers touching the object, this gesture changes the direction of the object to face the user. An example of changing the direction of an object by this gesture is shown in Figure 8.
- Copy object:
  On judging that two different users are each generating a touch point with respect to a single object, the system duplicates that object. Specifically, in the event that user B performs a single touch on an object while User A is performing a single touch on that object, the object will be copied and placed at the position of User B’s touch point. An example of the copy gesture is shown in Figure 9.

## 4. System Implementation

### 4.1. Tabletop

**Figure 10:**
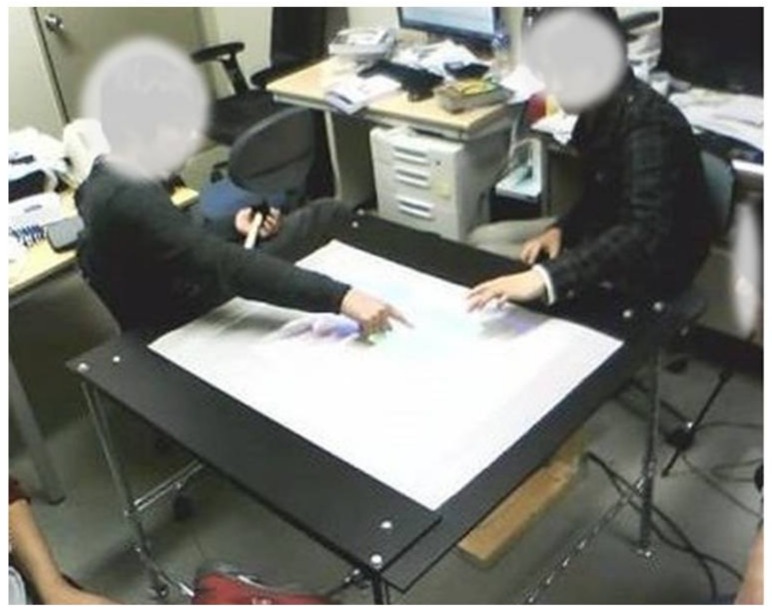
Tabletop system.

We created this system using Visual C and OpenCV running on Microsoft Windows. The FTIR-tabletop infrared floodlight consists of infrared LEDs and a control circuit. The frame rate of the infrared camera is 30 fps maximum. The tabletop itself is 70 cm high with a panel size of 100 cm × 90 cm and a display range of 60 cm × 50 cm. To fix a distance between the projector and the projection surface, we inserted a mirror between the table and projector. A typical scene of two users manipulating displayed objects using this tabletop system is shown in Figure 10.

**Figure 11:**
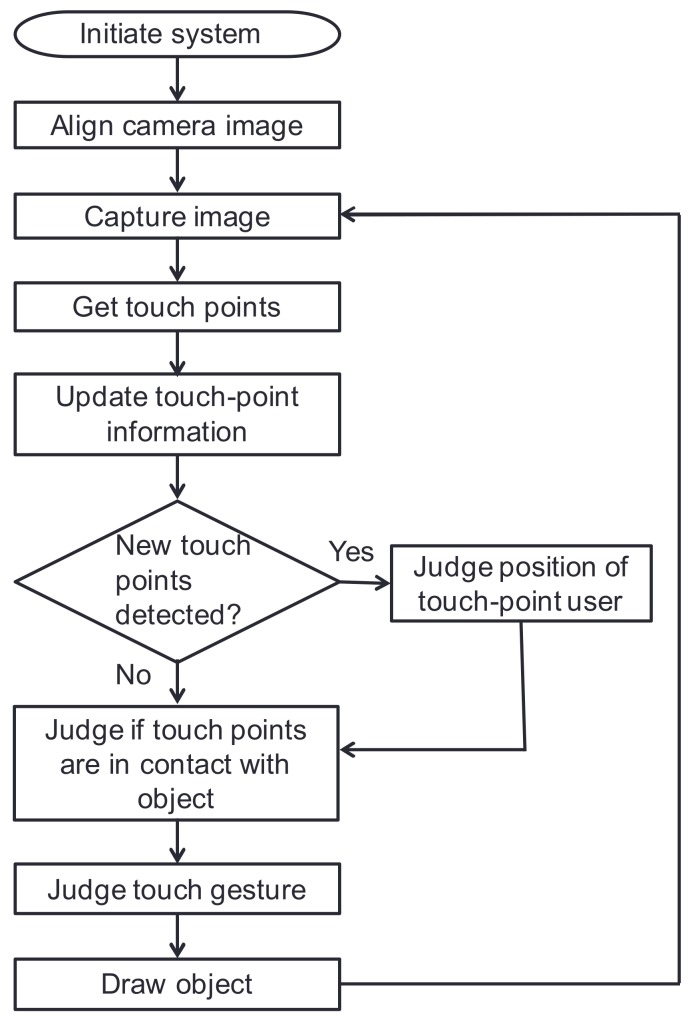
System procedure.

The procedure of this tabletop system is shown in Figure 11. In this system, the camera pickup area is set somewhat larger than the projector projection area and the rectangular projector projection area is cut out from the camera image to perform image alignment once. As explained in Section 3, obtaining touch points is accomplished by converting the background image and captured image to gray scale, performing difference calculations and threshold processing, removing noise, and extracting and labeling touch areas. Each touch area possesses certain types of information such as center-of-gravity coordinates and number of pixels.

Since touch points appear, move, and disappear by user touch operations, the system compares touch areas between the new and previous frame to update touch-point information. If the centers of gravity of touch areas having the same label should change between the previous frame and new frame, those touch points are judged to have moved. The process of determining the position of the user manipulating certain touch points follows the design presented in Section 3.

### 4.2. Photo-Object Manipulation Application

This system is equipped with an application for manipulating photo objects according to the touch gestures performed by multiple users. This photo-object manipulation application reads in image data as photo objects and displays them on the tabletop. The user manipulates objects by touch gestures.

## 5. Evaluation Experiments

We performed experiments with subjects to measure the recognition accuracy of the change-direction gesture and copy gesture and evaluate the usability of this system.

### 5.1. Experimental Setup

**Figure 12:**
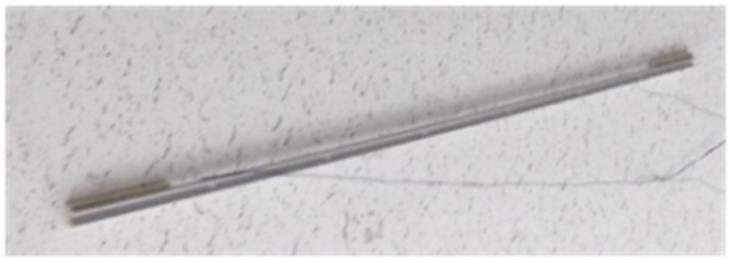
Infrared light.

**Figure 13:**
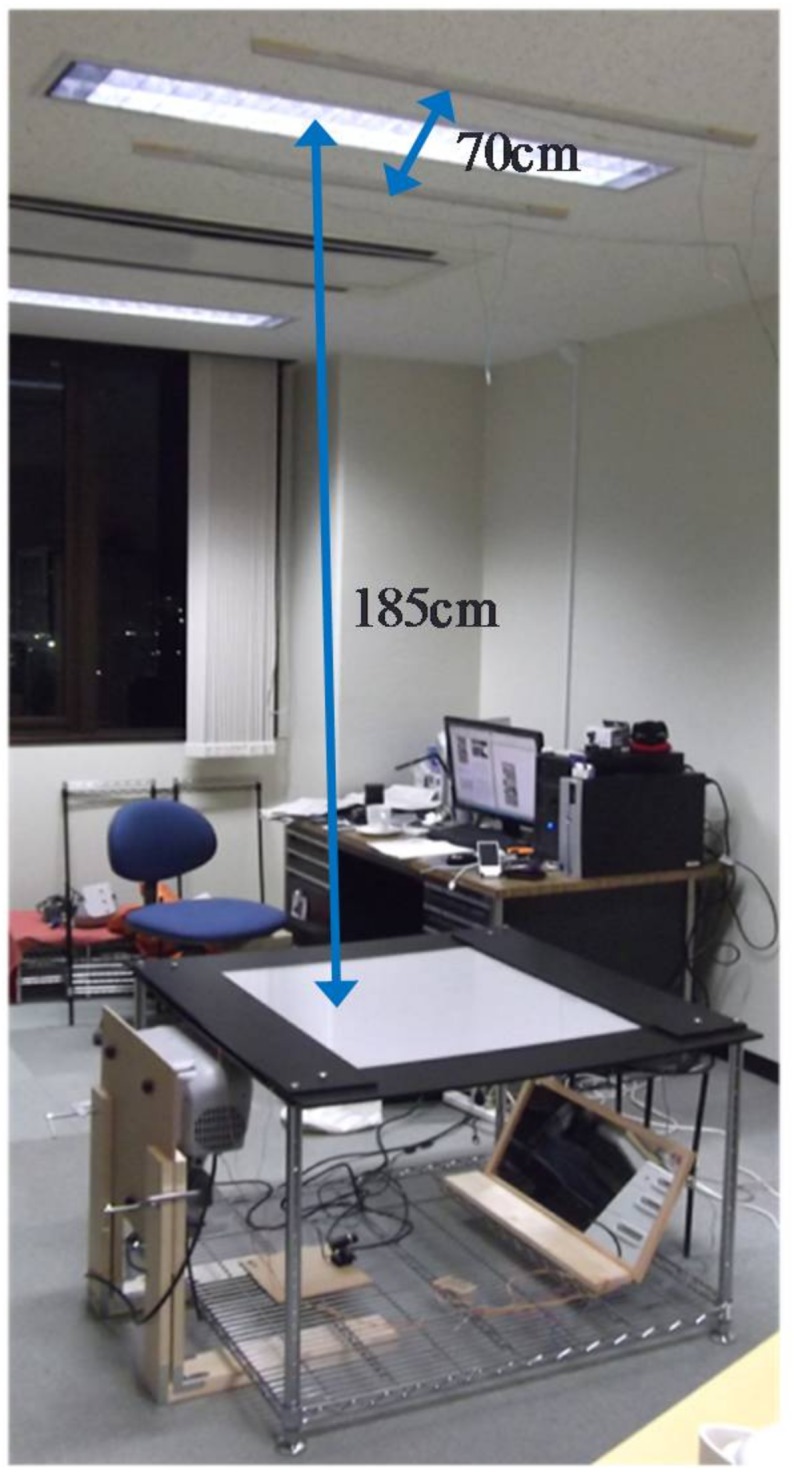
Experimental setup.

Taking into account the effects of sunlight on infrared light, we performed the experiments at night. We placed the prototype tabletop described in Section 4.1 in the center of the room and installed 2 infrared lights on the ceiling above the table, spacing them 70 cm apart. Each light was 90 cm long, incorporating 6 equally spaced infrared LEDs. We placed these infrared lights on either side of a fluorescent lamp on the ceiling. The distance from the ceiling to the tabletop panel was 185 cm. The infrared light is shown in Figure 12 and the experimental setup is shown in Figure 13.

### 5.2. Recognition Accuracy Experiment for Change-Direction Gesture

We performed a subject-based experiment for the 3-finger change-direction gesture and determined the identification rate of user position. Following an explanation of gesture operation, we asked each of 4 male subjects in their 20 s to perform the gesture operation 10 times on the tabletop system in each of 4 different directions. In the experiment, we compared the system-estimated and actual user positions and computed the user-position identification rate. Denoting the number of times the gesture was performed as *N_act_* and the number of times the system estimation agreed with the actual user position as *N_correct_*, the user-position identification rate was computed by Equation (10).

(10) $$\mathrm{Identification}\;\mathrm{rate}=\;\frac{N_{correct}}{N_{act}}\;\times 100\;\left[\%\right]$$

**Figure 14:**
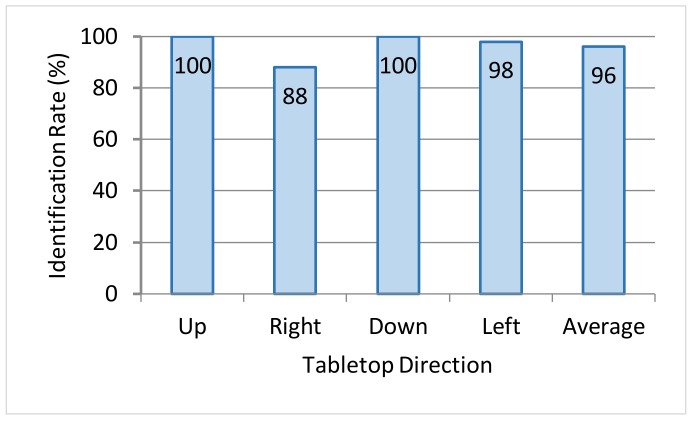
Identification rate for change-direction gesture.

For the sake of clarity, directions *d* = 1, 3 and directions *d* = 4, 2 in Figure 5 are called up/down directions and left/right directions, respectively. The average identification rate of the change-direction gesture from each of the 4 directions as performed by the 4 subjects is shown in Figure 14. The overall average identification rate of the change-direction gesture was approximately 96%. A broad classification of these results reveals that hand direction could be accurately identified in the up/down directions but that there were cases in which it could not be correctly identified in the left/right directions.

**Figure 15:**
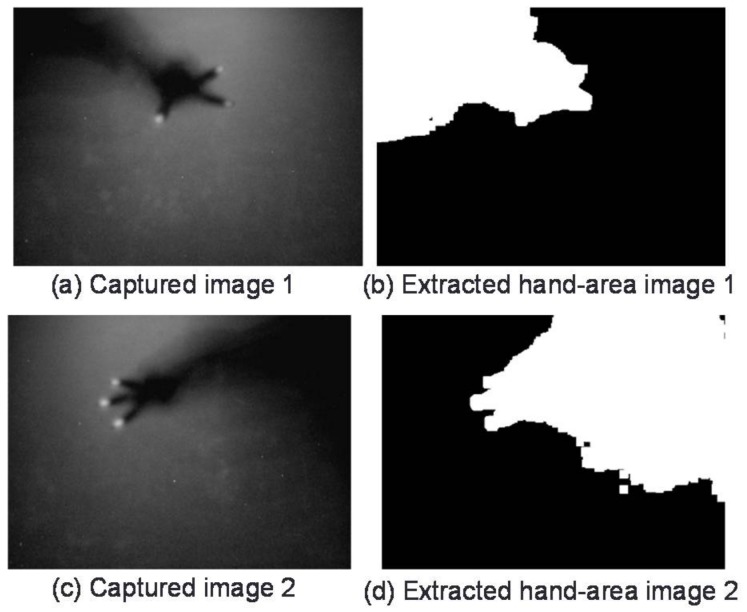
Change-direction gesture from left/right directions.

In this regard, we note that the left/right edges of the table were shorter than the up/down edges. The experimental results in Figure 14 indicate that when users positioned in the left/right directions manipulate an object situated near an up/down edge, there is a tendency for the hand shadow to cross that up/down edge. Examples in which left/right hand direction could not be correctly identified are shown in Figure 15. These examples show users performing the change-direction gesture from the left and right directions. In either case, the number of pixels in the hand-shadow area intersecting the upper edge is greater than those intersecting the left or right edge. As a result, the system erroneously judges the operation to be that of the user positioned in the up direction.

### 5.3. Recognition Accuracy Experiment for Copy Gesture

We performed a subject-based experiment for the copy gesture and measured its recognition rate. In this experiment, we divided 8 male subjects in their 20 s into 2 groups of 4 subjects each. With a subject positioned at each edge of the table, we had the subjects perform the copy gesture in 2 combinations—face-to-face across the table and side-by-side at neighboring edges—and measured the gesture recognition rate. Denoting the number of times the gesture was performed as *N_act_* and the number of times the system recognized the copy gesture as *N_detect_*, the recognition rate was computed by Equation (11).

(11) $$\mathrm{Recognition}\;\mathrm{rate}\;=\;\frac{N_{detect}}{N_{act}}\;\times 100\;\left[\%\right]$$

**Figure 16:**
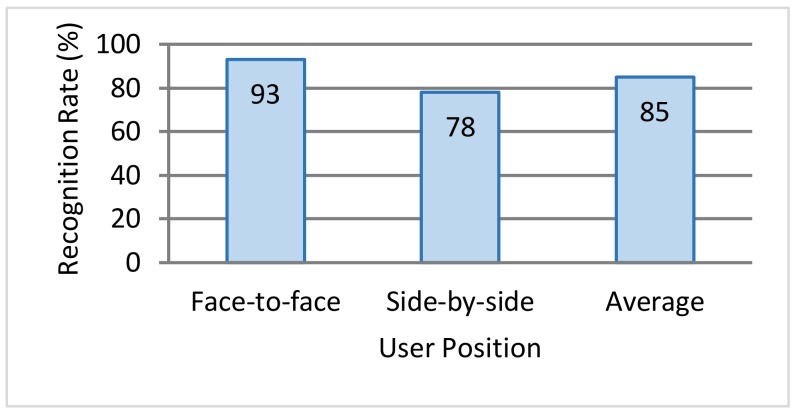
Recognition rate for copy gesture.

**Figure 17:**
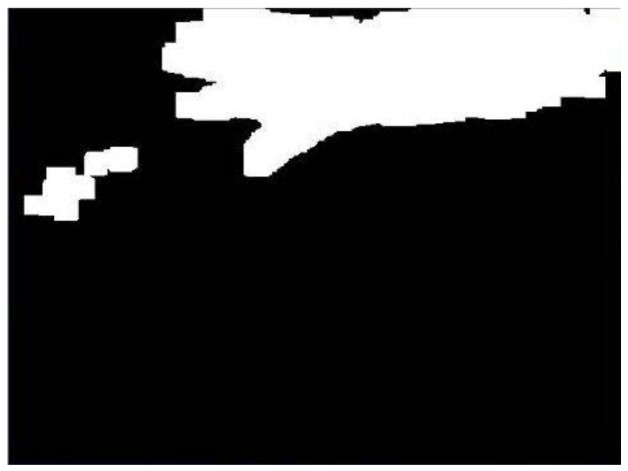
Example of failed recognition of a user’s direction.

The recognition rate for the copy gesture by 8 subjects is shown in Figure 16. The overall average recognition rate of the copy gesture was approximately 85%. Gesture recognition could fail here if the direction of 1 of the 2 users performing this gesture could not be identified. An example of failing to identify the direction of 1 of 2 users is shown in Figure 17. In this case, 2 face-to-face users in the left and right directions are performing the copy gesture. Given a 2-finger touch gesture, this system would judge the operation to be a copy gesture provided that the user direction of each touch point could be identified and judged to be different. In the example of Figure 17, the position of the user on the right side of the image could be identified from the user’s hand-shadow area. However, the head of the user on the left side created a shadow, and as a result, the hand-shadow area could not be distinguished from the dark portion of the background, preventing the direction of that user from being identified.

### 5.4. Results of System-Usability Evaluation and Discussion

After asking male subjects in their 20 s to freely use the system for about 5 min, we conducted a questionnaire-based survey using 4 subjects. This questionnaire consisted of questions based on the SUS evaluation method [20] and a section for open comments at the end. Ten questionnaire items based on the SUS evaluation method are given in the Appendix A.

**Figure 18:**
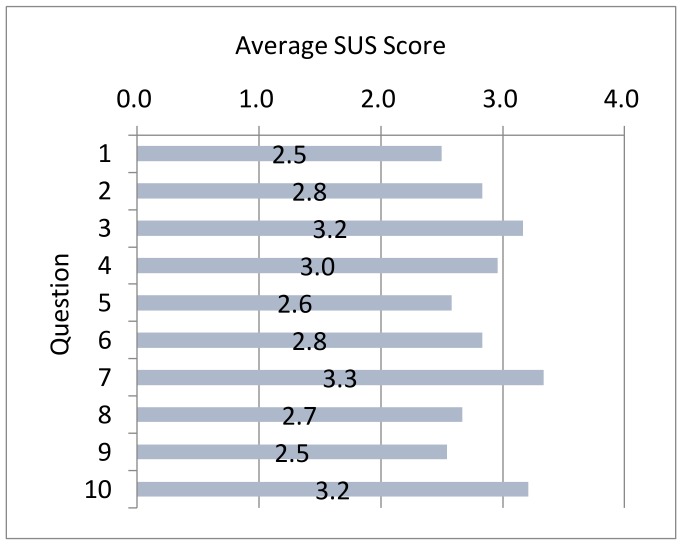
Average score for each item by SUS evaluation method.

In the survey, a subject responded to each questionnaire item on a 5-point scale. In tabulating scores, we followed the SUS evaluation method that subtracts 1 from the score of each odd-numbered item and subtracts the score of each even-numbered item from 5. The average score of each item out of 4 points based on the SUS evaluation method is shown in Figure 18.

In the SUS evaluation method, the scores for each questionnaire item are summed and then multiplied by 2.5 to convert to a 100-point scale. This value is taken to be the usability score. For the proposed system, the usability score by the SUS evaluation method was found to be 71.6 on average out of 100 points. In addition, a breakdown of questionnaire results revealed that items 3, 7, and 10 had high scores. These items are “3. I thought the system was easy to use”, “7. I would imagine that most people would learn to use this system very quickly”, and “10. There was no need to learn a lot of things before I could get going with this system”. Based on these results, the system was highly rated for making prior learning easy for the user and for being easy to operate.

In addition, the section for open comments included the statement, “How about showing the users the results of user identification”. This opinion implies an operation of detecting a hand before it touches the table surface, identifying the position of that user, and visually notifying the user of that result with a cursor. We consider that presenting the users with system recognition results in near real time in this way should improve system operability. On the other hand, comments such as, “Response of the move gesture is not so good”, and, “Trying to operate the system with two hands sometimes fails”. These comments reflect the need to improve system construction technology.

## 6. Conclusions

This paper described the development of a multi-touch tabletop system that identifies user position by infrared image recognition and presented the results of touch-gesture recognition accuracy experiments and a system-usability evaluation. The proposed system picks up touch points and the shadow area of a user’s hand by an infrared camera using an FTIR touch panel and infrared light and estimates the position of that user by image recognition. The multi-touch gestures prepared for this system include an operation to change the direction of an object and a copy operation in which two users generate duplicates of an object in addition to basic touch gestures.

With this system, the average recognition rates of the change-direction gesture and copy gesture were found to be 96% and 85%, respectively. The results of the questionnaire-based system-usability evaluation, meanwhile, revealed that prior learning was easy for the user and that system operations could be easily performed. At the same time, opinions expressed in the open-comments section of the questionnaire indicated that further improvements in system construction technology were needed for advanced interaction.

Future research topics include system improvement for further accurate identification, statistical analysis of several subject-based experiments, and enhancement of object-operation application functions, such as an object deletion function, a visual-support function, and gesture functions that make use of user position identification. Going forward, an important goal will be to combine the research of tabletop systems with other technologies and to associate such systems with application fields in great demand by society. For example, there is a high social need for combining tabletop systems with sensor technology in the field of medical treatment technology. In addition, the problem of occlusion that arises when irradiating people with light is not limited to systems using infrared light. It must also be kept in mind that infrared light is affected by sunlight. To solve or prevent these problems, further studies are needed to achieve and apply technical advances.

## Figures and Tables

**Table 1:** Touch gestures.

Operation	No. of Users	No. of Touches	Description
Move	1	1	Move object
Zoom in/out		2	Change object size
Rotate			Rotate object
Change direction		3	Change object’s direction to face user
Copy	2	2	Copy object

## Appendix A

*The System Usability Scale*: When an SUS is used, participants are asked to score the following 10 items with one of five responses that range from Strongly Agree to Strongly Disagree:

1. I think that I would like to use this system frequently.
2. I found the system unnecessarily complex.
3. I thought the system was easy to use.
4. I think that I would need the support of a technical person to be able to use this system.
5. I found the various functions in this system were well integrated.
6. I thought there was too much inconsistency in this system.
7. I would imagine that most people would learn to use this system very quickly.
8. I found the system very cumbersome to use.
9. I felt very confident using the system.
10. I needed to learn a lot of things before I could get going with this system.
